# Patients’ Satisfaction With Virtual Phone Clinics During the COVID-19 Pandemic at Outpatient Clinics in a Teaching Hospital, Riyadh, Saudi Arabia

**DOI:** 10.7759/cureus.48368

**Published:** 2023-11-06

**Authors:** Abdulaziz M Alrwais, Abdulmajeed F Alharbi, Nwaf Alwatban, Abdulaziz Alghufaili, Abdulrahman Alqasem, Abdulrahman Alroqi, Yousef A Alturki

**Affiliations:** 1 College of Medicine, King Saud University, Riyadh, SAU; 2 Family Medicine, College of Medicine, King Saud University, Riyadh, SAU

**Keywords:** covid 19, outpatients clinics, satisfaction, teaching hospital, virtual phone clinics

## Abstract

Introduction

The concept of virtual clinics, which have been in existence since the 1960s, was initially limited to military and space applications due to infrastructure limitations. However, with the evolution of communication technology and infrastructure improvements, virtual clinics became accessible to the general public, although they were not widely adopted until the coronavirus disease 2019 (COVID-19) pandemic. Virtual clinics offer several benefits, including overcoming distance-related challenges, providing healthcare services to underserved areas, reducing medical costs, and saving patients time. this research aims to assess patient satisfaction with virtual clinics in Saudi Arabia after the COVID-19 pandemic preventive measures are lifted, allowing for a comparison between virtual and traditional face-to-face clinics. This research aims to provide a more comprehensive assessment of patient satisfaction with virtual clinics.

Methods

We conducted a cross-sectional study and interviewed patients who had attended KKHU (King Khalid University Hospital) outpatient clinics during the COVID-19 pandemic to assess their level of satisfaction with virtual clinics through a questionnaire.

Results

The questionnaire was completed by 221 participants, 201 of whom met our inclusion criteria. The average score was 3.89, the standard deviation was 0.76, and the overall satisfaction ranged from 3.78 to 4. The mean and SD of the overall technical aspect satisfaction were 4.1 and 0.897, respectively. The mean and SD for the total perceived quality of care satisfaction were 3.89 and 0.95, respectively. The mean score for overall administration satisfaction was 3.74, and the SD was 0.92. The mean and SD for the virtual clinics as a whole were 3.82 and 0.73, respectively.

Conclusion

The overall level of satisfaction was 3.78 out of 5; however, 53% of participants did not want their next visit to be virtual and 73% of males and 63% of females were satisfied with KKHU outpatient clinics.

## Introduction

Coronavirus disease 2019 (COVID-19) is a newly discovered coronavirus that originated in Wuhan, China, in December 2019 [1]. The WHO has continuously assessed COVID-19, announcing that, as of March 11, 2020, it was a pandemic [2]. Saudi Arabia issued many measures to decrease its spread. For instance, on April 6, 2020, a 24-hour curfew was implemented in its capital, Riyadh [3]. As of December 10, 2022, the cumulative cases of COVID-19 were 826,153, with 9,482 deaths [4].

Virtual clinics are useful tools for overcoming distance-related challenges. For example, they can provide hospital services to physically disabled patients and patients living in rural, underserved areas, which was one of the main reasons for their implementation before the COVID-19 pandemic [5]. The accessibility of virtual clinics alone indicates their importance and significant potential. Nevertheless, they have more to offer than accessibility. Some studies have claimed they can reduce medical costs, although the evidence remains inconclusive [6,7], as few studies have considered their cost-effectiveness. Yet virtual clinics can save patients time because no traveling or commuting is involved, and there is negligible waiting time. Therefore, few patients are choosing virtual clinics over traditional face-to-face clinics [8].

There is some uncertainty revolving around the effectiveness, quality, and safety of telephone consultations [9], but a large-scale implementation of such a service would give more accurate insight and results of its efficacy.

In 2019, telephone consultations represented only 8% of medical appointments in the United States while between March 2 and April 14, 2020, telephone consultations increased by 683% [10]. Due to the increased number of users, assessing patient satisfaction is essential.

Previous studies in Saudi Arabia have shown various limitations. For example, a study undertaken in primary care clinics did not distribute the sample evenly between clinics [11]. Others lacked follow-up [12], or the sampling method altered the results [13]. Due to these limitations, a new study is encouraged to consider and limit such factors.

Our research aimed to assess the level of patients’ satisfaction with virtual clinics attended during the COVID-19 pandemic after precautionary measures for preventing the spread of COVID-19 were lifted in Saudi Arabia. The patients returning to traditional face-to-face clinics provided a means of comparing the two types of clinics.

## Materials and methods

A descriptive cross-sectional study design was conducted at King Khalid University Hospital (KKUH) outpatient clinics in Riyadh, Saudi Arabia, from June to December 2022. Patients who had attended a virtual clinic at least once during the COVID-19 pandemic were selected as the study population. Patients who did not attend a virtual clinic or had attended a virtual clinic before the COVID-19 pandemic were excluded from the study.

Data were collected using a questionnaire with 25 questions. The questionnaire was taken from previously published research [12]. After receiving the patient's consent, the data were collected in the KKUH outpatient clinic from early September to November 2022. The researcher distributed QR codes for the electronic questionnaire to participants who had attended KKUH’s outpatient clinics on random days (convenience sampling) after receiving consent and ensuring that the participants met the inclusion criteria.

The study was ethically approved on August 21, 2022, by the Institutional Review Board of King Saud University, with reference number E-22-7048 (B5 Group) CMED-305. Permission was obtained for distributing the QR codes to the participants in the KKUH outpatient clinic. Data were analyzed using IBM SPSS Statistical software for Windows version 21.0 (IBM Corp., Armonk, NY, USA). Descriptive statistics (frequencies and percentages) were used to describe the categorical variables, along with the mean, standard deviation, and a 95% confidence interval for the coded data. Pearson’s chi-square test was used to confirm the association during an analysis of the study data. The analyzed data were presented in tabular and graphic formats. The p-value and 95% confidence intervals ensured the precision of the results and determined their statistical significance.

After analyzing the age of participants by obtaining mean, standard deviation, and range, the participants were divided into four age groups: ≤25, 26-40, 41-60, and >60. The rest of the questionnaire was divided into four categories: technical aspects, perceived quality of care, administrative aspects, and an overall impression of virtual clinics. Each category assessed a different aspect of patient satisfaction with virtual clinics to show their strengths and weaknesses. There were five possible answers to each question in the various categories of the questionnaire. Each answer was assigned a value from 1 (not very unsatisfied) to 5 (very satisfied), with 3 indicating neutral. The four categories were assessed individually by obtaining the frequency, percentage, mean out of 5, standard deviation, and 95% confidence interval. Then, all the categories were combined to provide the final overall level of satisfaction of virtual clinic users.

Each participant was grouped into one of the five following groups based on the overall level of satisfaction: very satisfied (5-4.2), satisfied (4.19-3.4), neutral (3.39-2.6), unsatisfied (2.59-1.8), and very unsatisfied (1.79-1). Unfortunately, we could not form any association between the level of satisfaction and the variables in the questionnaire because of low responses in the “unsatisfied” and “very unsatisfied” groups, making it impossible to utilize any statistical test. However, we combined “unsatisfied” and “very unsatisfied” into one category and “satisfied” and “very satisfied” into another, resulting in three levels of satisfaction: unsatisfied, neutral, and satisfied. We could then use the chi-square test. We were able to obtain two associations between two study variables with the overall level of satisfaction but could not do so with the other variables.

## Results

A total of 221 participants filled out the questionnaire, 201 of whom met the inclusion criteria. The participants’ ages were normally distributed with a mean of 40.37 and a standard deviation (14) ranging between 16 and 85. The rest of the participants’ demographics and characteristics are shown in Table 1.

**Table 1:** Distribution of the socio-demographic characteristics of study subjects (= 201)

Characteristics	Frequency	Percentage (%)
Age*		
≤25	29	(14.4)
26–40	76	(37.8)
41–60	77	(38.3)
>60	18	(9)
Frequency of visits		
1–4	158	(78.9)
5–10	28	(13.9)
>10	15	(7.5)
Gender		
Male	154	(76.6)
Female	47	(23.4)
Marital status		
Single	52	(25.9)
Married	140	(69.7)
Divorced	9	(4.5)
Nationality		
Saudi	192	(95.5)
Non-Saudi	9	(4.5)
City		
Riyadh	174	(86.6)
Outside Riyadh	27	(13.4)
Level of education		
Lower than elementary school	4	(2)
Elementary school	4	(2)
Secondary school	9	(4.5)
Highschool	45	(22.4)
Diploma	21	(10.4)
College or higher	118	(58.7)
The reason for visiting a virtual clinic		
Regular follow-up	75	(37.3)
Discussion of symptoms	64	(31.8)
Discussion of treatment	32	(15.9)
Discussion of lab or radiology results	17	(8.5)
Other	13	(6.5)
Prefer the next visit to be a virtual clinic		
YES	94	(46.8)
NO	107	(53.2)
*n = 200, due to missing age data		

**Figure 1:**
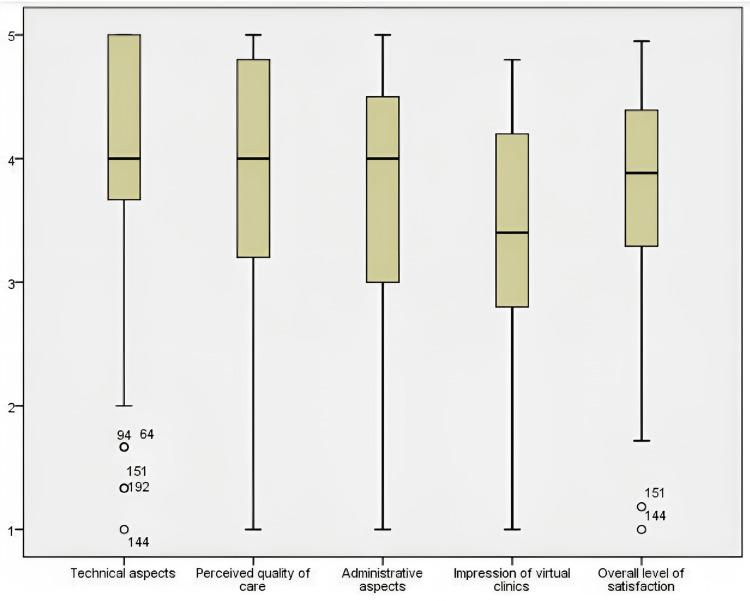
Mean level of satisfaction per category out of 5

The participants were asked about their levels of satisfaction with traditional and virtual clinics. The results for the traditional clinics were a mean of 4.33 out of 5 and a standard deviation of 1, compared to 4.01 and a standard deviation of 1.18 for virtual clinics. The details of the questionnaire results can be found in Tables 2-5. By calculating the mean of all four categories (technical, administrative, impression, and quality of care), we obtained the mean of the overall level of satisfaction (3.78) and the standard deviation (0.79), with a 95% CI (3.67-3.89). All four categories and the overall level of satisfaction are displayed in Figure 1.

**Table 2:** Distribution of patient satisfaction responses to the technical aspects of virtual clinics (n=201) 1 = strongly disagree, 2 = disagree, 3 = neutral, 4 = agree, 5 = strongly agree

Item of Satisfaction	Frequency (%)					Mean (SD)
	5	4	3	2	1	
I could hear my doctor clearly.	97 (48.4)	71 (35.3)	14 (7)	14 (7)	5 (2.5)	4.2 (1.01)
There was no significant lag in the sound.	93 (46.3)	71 (35.3)	25 (12.4)	9 (4.5)	3 (1.5)	4.2 (.929)
I am satisfied overall with the technical aspects of virtual clinics.	73 (36.3)	72 (35.8)	31 (15.4)	16 (8)	9 (4.5)	3.92 (1.11)
				4.1 (.897), 95% CI (3.98–4.23)		

**Table 3:** Distribution of patient satisfaction responses to the administrative aspects of virtual clinics (n=201) 1 = strongly disagree, 2 = disagree, 3 = neutral, 4 = agree, 5 = strongly agree

Item of Satisfaction	Frequency (%)					Mean (SD)
	5	4	3	2	1	
The appointment was easily scheduled.	64 (31.8)	87 (43.3)	29 (14.4)	13 (6.5)	8 (4)	3.93 (1.03)
I know what to expect during a virtual clinic.	44 (21.9)	67 (33)	60 (29.9)	19 (9.5)	11 (5.5)	3.57 (1.09)
Overall administrative aspects				3.74 (.92) 95% CI (3.61–3.87)		

**Table 4:** Distribution of patient satisfaction responses to perceived quality of care received in virtual clinics (n= 201) 1 = strongly disagree, 2 = disagree, 3 = neutral, 4 = agree, 5 = strongly agree

Item of Satisfaction	Frequency (%)					Mean (SD)
	5	4	3	2	1	
I felt relaxed while interacting with my physician.	68 (33.8)	68 (33.8)	37 (18.4)	21 (10.4)	7 (3.5)	3.84 (1.11)
I felt comfortable discussing and addressing all of my concerns.	69 (34.3)	71 (35.3)	32 (15.9)	20 (10)	9 (4.5)	3.85 (1.13)
I felt understood by my physician during the virtual (phone) clinic.	66 (32.8)	75 (37.3)	27 (13.4)	24 (11.9)	9 (4.5)	3.82 (1.14)
I felt that there was enough time to address my concerns.	64 (31.8)	61 (30.3)	38 (18.9)	29 (14.4)	9 (4.5)	3.71 (1.18)
I felt that my privacy was respected.	93 (46.3)	81 (40.3)	19 (9.5)	4 (2)	4 (2)	4.27 (.865)
Overall perceived quality of care received				3.89 (.95) 95% CI (3.76 – 4)		

**Table 5:** Distribution of patient satisfaction responses to the impression of virtual clinics (n=201) 1 = strongly disagree, 2 = disagree, 3 = neutral, 4 = agree, 5 = strongly agree

Item of Satisfaction	Frequency (%)					Mean (SD)
	5	4	3	2	1	
I would use a phone clinic again.	59 (29.4)	65 (32.3)	34 (16.9)	28 (13.9)	15 (7.5)	3.62 (1.24)
It is easier for me to talk with my healthcare provider on the phone than face-to-face care.	57 (28.4)	50 (24.9)	34 (16.9)	39 (19.4)	21 (10.4)	3.41 (1.35)
I prefer to see my physician in person rather than via a phone clinic.	101 (50.2)	44 (21.9)	39 (19.4)	16 (8)	1 (.5)	1.87 (1.02)
virtual phone clinic saves me time during the day to do other tasks.	86 (42.8)	73 (36.3)	24 (11.9)	13 (6.5)	5 (2.5)	4.1 (1.01)
virtual phone clinic makes me less dependent on others.	81 (40.3)	55 (27.4)	36 (17.9)	17 (8.5)	12 (6)	3.88 (1.2)
Overall Impression of virtual (phone) clinics				3.37 (.88), 95% CI (3.25–3.49)		

Table 6 shows the association between the level of satisfaction with gender and next-visit preference, which was determined using the chi-square test. Males indicated a higher, but statistically insignificant, level of satisfaction than females. The question about a preference for a subsequent visit revealed, with a high level of statistical significance, that 54.2% of those who favored regular clinics were satisfied with virtual clinics.

**Table 6:** The association between the overall level of satisfaction with gender and next-visit preference (n=201)

		Overall Level of Satisfaction				
		Satisfied (%)	Neutral (%)	Unsatisfied (%)	Total	Chi-square (p-value)
Gender	Male	113 (73.3)	30 (19.4)	11 (7.1)	154	2.93 (.23)
	Female	30 (63)	10 (21.2)	7 (14.8)	47	
I prefer the next visit to be virtual	Yes	85 (90.4)	9 (9.5)	0 (0)	94	34.5
	No	58 (54.2)	31 (28.9)	18 (16.8)	107	

## Discussion

The study was conducted on a sample from inside and outside the city of Riyadh who had attended KKUH, with percentages of 86.6% and 13.4%, respectively. Technical aspects yielded the highest score (4.1) among the other questionnaire categories in our study, which can be helpful for us to eliminate dissatisfaction due to technical issues to an extent, it is a step up from some earlier research [14,15]. the technological issues can easily be solved later with a few updates, giving us a better understanding of the true level of satisfaction [12]. A Saudi Arabian study that employed the same questionnaire as our study and was conducted in a different city discovered higher satisfaction with technical features in general (4.53) [12]. Compared to the values for [12], which were 4.73 and 4.37, respectively, the perceived quality of care and administrative elements received scores of 3.89 and 3.74, respectively.

The overall impression of virtual clinics scored the lowest level of satisfaction (3.37), possibly because few questions in the category compared virtual clinics with traditional clinics, indicating that people still find traditional clinics more appealing. This trend continued in the question about next-visit preference, as 53% of participants did not want their next visit to be virtual. However, 54% of those were actually satisfied with virtual clinics, and only 16% were unsatisfied, perhaps indicating that virtual clinics have obstacles that must be identified and overcome. Despite that, other studies have shown that even after COVID-19, healthcare professionals and patients have expressed interest in using telemedicine as part of their regular follow-up visits. A study titled “Gastroenterologists and Patients Report High Satisfaction Rates with Telehealth Services During the Novel Coronavirus 2019 Pandemic” discovered that virtual clinics were quite satisfying (4.28) [12,16]. In research by Firas Hentati, 62.2% of participants said they preferred in-person visits over telemedicine consultations [17]. As opposed to new patient contacts, follow-up, and postoperative visits were linked to better patient satisfaction levels in a study by Janet S. Choi [18]. The findings of a study by Bhuva S show that telehealth can be a tool to deliver satisfactory and effective care, particularly with follow-up visits, in which more patients value a virtual consultation over a face-to-face meeting [19]. The overall level of satisfaction in our study was 3.78, which was significantly lower than [12], which reported a level of 4.49.

There was no statistically significant association between gender and level of satisfaction. However, 73% of males were satisfied with virtual clinics as compared to 63% of females but Alharbi demonstrated a statistically significant gender effect on patient satisfaction that favored men (p=0.003) [11].

Our study had two limitations. First, the sample was small. Second, most participants were men because not enough women used telephone clinics.

We recommend further national studies with larger sample sizes in different health institutions. These studies should explore other factors that may play a role in virtual clinic adoption. They should also determine patients’ satisfaction with phone clinics in other areas, especially rural regions.

## Conclusions

Virtual clinics have become prominent and widely used, especially after the COVID-19 pandemic, when they were established to protect patients and physicians. The study showed that virtual telephone clinics could be implemented in hospitals and health centers with a significant level of patient satisfaction. Although many patients did not want future clinics to be virtual following the COVID-19 pandemic, healthcare centers should increase their efforts and offer virtual telephone visits so that patients can become accustomed to them.
